# Soluble neprilysin, NT-proBNP, and growth differentiation factor-15 as biomarkers for heart failure in dialysis patients (SONGBIRD)

**DOI:** 10.1007/s00392-020-01597-x

**Published:** 2020-01-30

**Authors:** Robert Claus, Dominik Berliner, Udo Bavendiek, Nicolas Vodovar, Ralf Lichtinghagen, Sascha David, Margret Patecki, Jean-Marie Launay, Johann Bauersachs, Hermann Haller, Marcus Hiss, Michael S. Balzer

**Affiliations:** 1 Department of Nephrology and Hypertension, Hannover Medical School, Carl-Neuberg-Str. 1, 30625 Hannover, Germany; 2 Department of Cardiology and Angiology, Hannover Medical School, Hannover, Germany; 3 INSERM UMR S-942, Hôpital Lariboisière, Paris, France; 4 INI-CRCT (Cardiovascular and Renal Clinical Trialists) F-CRIN Network, Nancy, France; 5 Institute of Clinical Chemistry, Hannover Medical School, Hannover, Germany; 6 Center for Renal, Hypertensive and Metabolic Disorders, Hannover, Germany

**Keywords:** Congestive heart failure (HF), Biomarker, Chronic kidney disease (CKD), Hemodialysis (HD), Peritoneal dialysis (PD), Neprilysin (NEP), Growth differentiation factor-15 (GDF-15)

## Abstract

**Background:**

Dialysis patients are at increased risk of HF. However, diagnostic utility of NT-proBNP as a biomarker is decreased in patients on dialysis. GDF-15 and cNEP are biomarkers of distinct mechanisms that may contribute to HF pathophysiology in such cohorts. The aim of this study was to determine whether growth differentiation factor-15 (GDF-15) and circulating neprilysin (cNEP) improve the diagnosis of congestive heart failure (HF) in patients on dialysis.

**Methods and results:**

We compared circulating concentrations of NT-proBNP, GDF-15, and cNEP along with cNEP activity in patients on chronic dialysis without (*n* = 80) and with HF (*n* = 73), as diagnosed by clinical parameters and post-dialysis echocardiography. We used correlation, linear and logistic regression as well as receiver operating characteristic (ROC) analyses. Compared to controls, patients with HF had higher median values of NT-proBNP (16,216 [interquartile range, IQR = 27739] vs. 2883 [5866] pg/mL, *p* < 0.001), GDF-15 (7512 [7084] vs. 6005 [4892] pg/mL, *p* = 0.014), but not cNEP (315 [107] vs. 318 [124] pg/mL, *p* = 0.818). Median cNEP activity was significantly lower in HF vs. controls (0.189 [0.223] vs. 0.257 [0.166] nmol/mL/min, *p* < 0.001). In ROC analyses, a multi-marker model combining clinical covariates, NT-proBNP, GDF-15, and cNEP activity demonstrated best discrimination of HF from controls (AUC = 0.902, 95% CI 0.857–0.947, *p* < 0.001 vs. base model AUC = 0.785).

**Conclusion:**

We present novel comparative data on physiologically distinct circulating biomarkers for HF in patients on dialysis. cNEP activity but not concentration and GDF-15 provided incremental diagnostic information over clinical covariates and NT-proBNP and may aid in diagnosing HF in dialysis patients.

**Graphic abstract:**

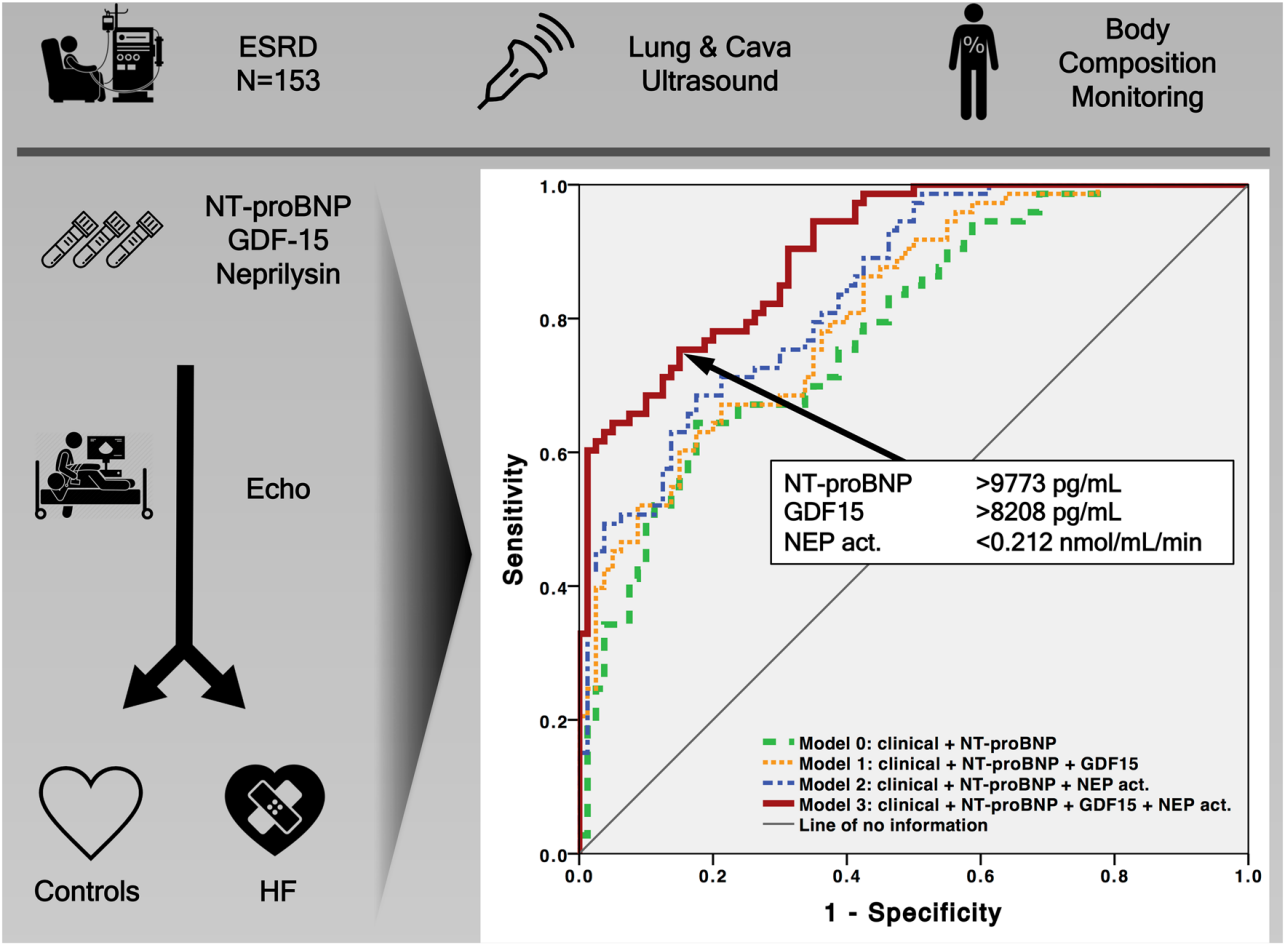

**Electronic supplementary material:**

The online version of this article (10.1007/s00392-020-01597-x) contains supplementary material, which is available to authorized users.

## Introduction

Cardiovascular morbidity and mortality are increased considerably in patients with chronic kidney disease (CKD) and those who progress to end-stage renal disease (ESRD) [1]. More than a third of ESRD patients initiating dialysis has congestive heart failure (HF), a risk which is up to 36 times higher than in the general population [2]. Another 25% develop de novo HF while on dialysis [3]. One risk factor for de novo HF is reduced left ventricular ejection fraction (LVEF), thus leading to increased mortality [4]. While measurement of natriuretic peptides for the diagnosis of HF has been a major landmark in cardiology [5], diagnostic utility of peptides such as N-terminal pro-B-type natriuretic peptide (NT-proBNP) as biomarkers for HF in ESRD patients is strongly reduced [6]. Excessively high NT-proBNP levels in ESRD patients without HF are due to both decreased renal elimination and increased prevalence of volume overload, hypertension, and consecutive left ventricular hypertrophy (LVH) [7]. NT-proBNP is mostly eliminated by glomerular filtration [8], explaining the strong influence of renal function on NT-proBNP concentrations. We therefore sought for a combination of biomarkers of distinct mechanisms in HF pathophysiology to increase utility for HF diagnosis in patients with ESRD.

Growth differentiation factor-15 (GDF-15) belongs to the transforming growth factor-β cytokine family. It is elevated due to multiple pathologic states including inflammation, oxidative stress, hypoxia, telomere erosion, and oncogene activation [9, 10]. In contrast to natriuretic peptides, it is not influenced by volume status [11]. Unlike NT-proBNP, GDF-15 levels are not increased in patients with atrial fibrillation [12] or decreased in obese patients [13]. In non-dialysis-dependent CKD patients, GDF-15 release is augmented and predicts mortality and risk of HF [14]. To the best of our knowledge, there is no information on the predictive value of GDF-15 for HF in patients with ESRD.

Neprilysin (NEP) is an endo-peptidase that degrades natriuretic peptides. Hence, it is increasingly being used as both diagnostic marker and target in HF therapy [15]. Furthermore, HF with reduced EF (HFrEF) patients receiving a combined angiotensin receptor and NEP inhibitor had lower cardiovascular death rates than patients treated with angiotensin-converting enzyme inhibitors (ACE-I) [16]. Some studies investigated the prognostic value of circulating NEP (cNEP) as a predictor for mortality and morbidity in HF [17, 18]. It is known to be associated with adverse cardiovascular events in HFrEF and acute decompensated heart failure [17] whilst this was not the case for patients with HF with preserved EF (HFpEF) [18]. cNEP is less impaired by decreased renal function and other comorbidities than NT-proBNP [19]. In patients with CKD stages 2–4, reduced cNEP activity but not concentration predicted future hospitalization for HF [20]. Finally, cNEP has not been studied as a diagnostic marker for HF in ESRD patients.

We hypothesized that using GDF-15 and cNEP as markers of distinct mechanisms in addition to NT-proBNP would yield increased performance for HF diagnosis in ESRD patients.

## Patients and methods

### Study population

The study was registered with clinicaltrials.gov (NCT04061811). A total of 153 patients from two outpatient dialysis clinics and one in-hospital dialysis facility were consecutively enrolled (Aug/2018–Feb/2019). To be eligible for the study, a patient had to be on either chronic hemodialysis (HD) or peritoneal dialysis (PD) for ≥ 3 months. Patients who had previously switched the type of renal replacement therapy from HD to PD or vice versa were excluded. Other exclusion criteria were age < 18 years and pregnancy. To minimize confounding of circulating biomarkers, patients who had received plasma exchange or apheresis in the past 6 months were excluded. To facilitate unbiased bioelectrical impedance analysis (BIA), patients with unipolar pacemaker and history of whole extremity amputation were excluded. Written informed consent was obtained from all study participants.

### Biochemical measurements

Venous serum and EDTA plasma samples were collected from all patients immediately before evaluation of volume status and echocardiography. To correct for changes in inter-dialytic volume status and to avoid confounding of biomarkers by volume overload, we collected blood samples from all patients receiving intermittent dialysis therapy immediately after the respective session. In a preliminary study, measurement of NT-proBNP, cNEP concentration, and cNEP activity demonstrated moderate to good correlation of samples analyzed pre- and post-HD. Where feasible, peritoneal dialysis fluid was sampled simultaneously. Serum and plasma were put on ice and centrifuged (3500RPM, 10 min, 4 °C). Blood and peritoneal fluid samples were stored at − 80 °C until further analysis. Concentrations of NT-proBNP and GDF-15 were measured in serum and peritoneal fluid using commercially available electro-chemiluminescence immunoassays (Roche, Basel, Switzerland) that were performed on a cobas e801 modular analytics system (Roche) [21]. cNEP (EC3.4.24.11) concentration was measured in plasma and peritoneal fluid using the SEB785Hu ELISA kit (USCN Life Science, Wuhan, China), and cNEP activity in plasma and peritoneal fluid was determined by fluorometry as previously described [22]. All other biochemical parameters were measured with routine laboratory methods.

### Evaluation of volume status

For each patient enrolled in the study, we performed a thorough evaluation of volume status through clinical examination, lung ultrasound, inferior vena cava diameter measurement, and BIA. For all HD patients and for PD patients on intermittent in-center regimens, information was obtained immediately after a random dialysis session. For PD patients with continuous dialysis delivery, evaluation took place as feasible for outpatient clinic routine. For all patients, greatest care was taken that asservation of blood samples, evaluation of volume status, and echocardiography were performed in immediate sequence and most timely fashion.

We obtained information on presence/absence of peripheral edema, crackles on lung auscultation, dyspnea, inter-dialytic body weight gain, net ultrafiltration volume, and pre- and post-dialytic systolic and diastolic arterial blood pressure. To evaluate crackles, we used the following scale (adapted from Kataoka and Matsuno [23]): 1, no crackles; 2, uncertain about the presence of fine crackles; 3, definite fine crackles at lung bases; 4, moderate crackles; and 5, bilateral, diffuse crackles. For clinical edema, the following scale was used: 1, no clinical edema; 2, slight pitting (2 mm depth) with no visible distortion; 3, somewhat deeper pit (4 mm) with no readily detectable distortion; 4, noticeably deep pit (6 mm) with the dependent extremity full and swollen; and 5, very deep pit (8 mm) with the dependent extremity grossly distorted.

Lung ultrasound was performed as described previously by the LUST investigators [24]. Shortly, we screened for ultrasound-B lines at 28 standardized intercostal positions on both sides of the chest. Inferior vena cava diameter was measured at the non-forced end-expiratory phase within the subxiphoid window of the inferior vena cava–right atrial junction.

Finally, BIA was performed using a body composition monitor according to the manufacturer’s instructions (Fresenius Medical Care, Bad Homburg, Germany) and only measurements adhering to strict quality control criteria were used. Data were extracted using Fluid Management Tool software v3.3. Total body water (TBW), intracellular water (ICW), extracellular water (ECW) as well as nutritional parameters were evaluated. We corrected ECW values for TBW (ECW:TBW ratio).

### Use of echocardiography for diagnosis of congestive heart failure

A diagnosis of HF was made on the basis of clinical together with echocardiography findings. The echocardiographic outcome parameter was the composite of either impaired systolic and/or diastolic dysfunction. All studies were acquired following the actual recommendations. Comprehensive echocardiography was performed immediately after BIA using standardized equipment (Philips EPIQ7 with X5-1 transducer) and adhering to a uniform image acquisition protocol. At the end of the echocardiographic record, systolic and diastolic function were evaluated by the cardiologist performing the echocardiography. All echocardiographic data was graded by a second cardiologist who was blinded to the clinical data of the patients. If the adjudicators disagreed, the second adjudicator overruled the first one. LVEF was assessed using the biplane method of disks (modified Simpson’s method). Patients with LVEF < 50% were classified as having systolic dysfunction. Presence of LVH was evaluated using interventricular septum thickness at end-diastole (IVSED), LV end-diastolic diameter (LVEDD), LV posterior wall thickness at end-diastole (LVPWD) to calculate LV mass. LV mass was indexed by body surface area (LVMI). The ratio of early transmitral flow to early mitral annular diastolic velocity (*E*/*eʹ*) was recorded as an index of LV filling pressure. LV diastolic dysfunction was rated using left atrial volume index (LAVI), velocity of early to late transmitral inflow (*E*/*A*), and *E*/*e*ʹ following the actual recommendations [25]. If analysis of diastolic function was inconclusive, patients were graded as not having diastolic dysfunction.

### Statistical analysis

We used IBM SPSS-Statistics v22.0 for data analysis. All tests were two-tailed. *P* < 0.05 was considered to indicate statistically significant differences. Categorical variables were compared among diagnostic groups using cross-tabulation, continuous variables were summarized by means ± standard deviation (SD) unless stated otherwise. D’Agostino and Pearson omnibus test was used to test for normality. All biomarkers were naturally log-transformed to reduce the effects of distribution skewness. Log-transformed biomarker distributions were standardized to mean = 0 ± 1 SD within sex to account for sex-related differences and to facilitate comparison of effect sizes between biomarkers. *T* tests, ordinary one-way ANOVA, Kruskal–Wallis and Mann–Whitney *U* tests were used for comparison of means as applicable.

Univariate Pearson’s or Spearman’s correlation was used to relate clinical, BIA, and echocardiographic covariates to biomarkers. Multiple linear regression analysis was used to determine covariates independently associated with biomarkers. We adjusted for the following clinical covariates: age, sex, systolic blood pressure, type of renal replacement therapy, dialysis vintage, net ultrafiltration, clinical volume status score, vena cava inferior diameter, lung comet score, ECW:TBW ratio, Charlson comorbidity index, NYHA class, coronary artery disease, valve disease, hypertension, and drugs such as angiotensin-converting enzyme inhibitors, beta blockers, oral anticoagulants, nitrates, and erythropoietin-stimulating agents.

For biomarker discrimination between congestive heart failure and controls, unadjusted and adjusted associations of log-transformed, sex-standardized biomarker levels with outcome (diagnosis of HF by clinical parameters and echocardiography) were evaluated by univariate and multivariate logistic regression models. Using backward selection, only significant variables (*p* < 0.05) were kept in the final model and all factors were tested for interaction with biomarkers. NT-proBNP, GDF-15, and cNEP activity were retained in the model, while cNEP concentration was not. Finally, we used different combinations of the retained biomarkers to construct weighted multi-marker scores as described previously [26]. In short, the sum of sex-standardized log-biomarker concentration weighed by the estimated regression coefficients of the respective biomarker constituted the risk score on a continuous scale. The risk score was used as a continuous predictor in SD units. Clinical covariates + NT-proBNP was used as base model (model 0) for prediction. Prognostic utility was evaluated by Harrell’s C-statistic. Incremental prognostic utility of GDF-15 and cNEP activity was assessed by comparing the areas under the curve (AUC) of receiver operating characteristics (ROC) curves after the addition of either GDF-15 (model 1) or cNEP activity (model 2) or both GDF-15 and cNEP activity (model 3) to the AUC of the base model (model 0). Analyses after exclusion of patients with atrial fibrillation yielded similar results.

## Results

### Baseline characteristics

Our study sample consisted of 73 patients with HF (*n* = 33 LVEF ≥ 50%, *n* = 24 40–49%, *n* = 16 < 40%) and 80 controls without HF. Baseline characteristics of the study sample are shown in Table 1 and Supplementary Table 1. HF patients were considerably older, had more comorbidities, and demonstrated more volume overload: they had higher edema, lung comet and dyspnea scores, a higher ECW:TBW ratio, and required more net ultrafiltration on dialysis to reach their dry weight. Moreover, remaining post-dialysis overhydration was more pronounced in HF patients than controls.

**Table 1:** Patient characteristics

Characteristic	Controls (*N* = 80)	HF (*N* = 73)	*P*
Age, years (mean ± SD)	56.6 ± 18.2	64.3 ± 16.2	**0.008**
Gender, male (%)	52 (65.0)	43 (58.9)	0.505
Height, cm (mean ± SD)	171.5 ± 10.7	169.8 ± 11.1	0.356
Weight, kg (mean ± SD)	77.2 ± 20.8	72.7 ± 16.4	0.161
BMI, kg/m^2^ (mean ± SD)	26.1 ± 5.8	25.1 ± 4.5	0.257
BSA, m^2^ (mean ± SD)	1.90 ± 0.30	1.84 ± 0.25	0.179
Heart rate, bpm (mean ± SD)	75.7 ± 13.0	74.7 ± 15.4	0.455
Systolic BP^a^, mmHg (mean ± SD)	122.7 ± 24.2	131.3 ± 24.8	0.051
Diastolic BP^a^, mmHg (mean ± SD)	70.5 ± 17.6	71.7 ± 14.1	0.498
Renal replacement therapy, *n* (%)			
HD	54 (67.5)	53 (72.6)	0.597
PD	26 (32.5)	20 (27.4)	
HD characteristics			
Dialysis access, *n* (%)			0.458
Fistula	46 (85.2)	42 (79.2)	
Catheter	8 (14.8)	11 (20.8)	
Systolic BP pre-HD (mean ± SD)	131.4 ± 22.2	136.0 ± 24.9	0.236
Delta pre–post-HD systolic BP (mean ± SD)	13.7 ± 19.4	8.8 ± 16.9	0.227
Interdialytic weight gain, g (mean ± SD)	1497 ± 1088	1443 ± 1016	0.987
Blood flow rate, ml/min (mean ± SD)	243 ± 25	246 ± 35	0.985
Ultrafiltration rate, mL/h/kg (mean ± SD)	4.7 ± 3.4	4.8 ± 3.3	0.664
Net ultrafiltration, mL (mean ± SD)	1451 ± 1069	1393 ± 994	1.000
PD characteristics			
Regimen, *n* (%)			0.364
APD	14 (53.8)	14 (70.0)	
CAPD	12 (46.2)	6 (30.0)	
Ultrafiltration rate, mL/h/kg (mean ± SD)	0.3 ± 0.3	0.6 ± 0.4	**0.045**
Net ultrafiltration, mL (mean ± SD)	593 ± 398	954 ± 562	**0.026**
Dialysis vintage, months (mean ± SD)	47.8 ± 49.7	67.0 ± 73.0	0.223
Volume status score, 2–10, *n* (%)			
2	51 (63.7)	35 (47.9)	0.202
3	19 (23.8)	21 (28.8)	
4	8 (10.0)	13 (17.8)	
5	2 (2.5)	4 (5.5)	
Auscultation score, 1–5, *n* (%)			
1	67 (83.8)	54 (74.0)	0.257
2	10 (12.5)	14 (19.2)	
3	2 (2.5)	5 (6.8)	
4	1 (1.3)	0 (0.0)	
Edema score, 1–5, *n* (%)			
1	62 (77.5)	43 (58.9)	**0.045**
2	13 (16.3)	25 (34.2)	
3	4 (5.0)	5 (6.8)	
4	1 (1.3)	0 (0.0)	
Vena cava inferior diameter, mm (mean ± SD)	13.8 ± 5.2	15.4 ± 4.59	0.056
Lung comet score (mean ± SD)	10.7 ± 20.7	16.6 ± 19.3	**0.003**
Dyspnea score, *n* (%)			
1	51(63.8)	31 (42.5)	**0.040**
2	14 (17.5)	21 (28.8)	
3	12 (15.0)	13 (17.8)	
4	3 (3.8)	8 (11.0)	
NT-proBNP, pg/mL (mean ± SD)	7345 ± 16,105	28,063 ± 42,295	< **0.001**
GDF-15, pg/mL (mean ± SD)	6822 ± 4365	9357 ± 7023	**0.020**
cNEP concentration, pg/mL (mean ± SD)	321 ± 120	321 ± 114	0.927
cNEP activity, nmol/mL/min (mean ± SD)	0.288 ± 0.142	0.212 ± 0.131	**0.001**

^a^For HD patients, blood pressure was measured post dialysis

*APD* automated peritoneal dialysis, *BP* blood pressure, *CAPD* continuous ambulatory peritoneal dialysis, *BSA* body surface area, *cNEP* circulating neprilysin, *GDF-15* growth differentiation factor-15, *HD* hemodialysis, *HF* congestive heart failure, *NT-proBNP* N-terminal pro-B type brain natriuretic peptide, *PD* peritoneal dialysis

### Biomarkers in patients with and without congestive heart failure

**Fig. 1:**
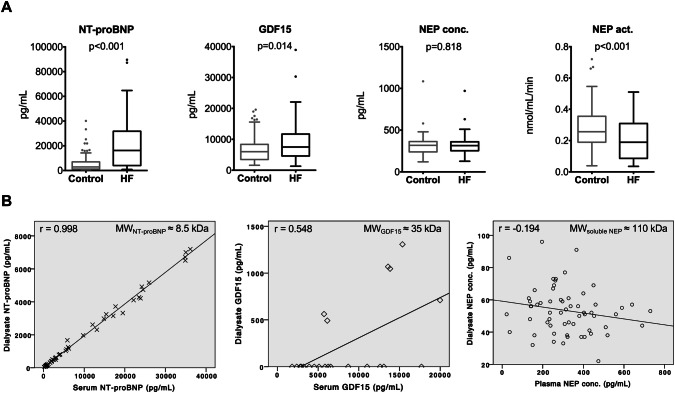
Biomarkers in patients with HF and controls. **a** The graph shows Tukey boxplots, *p* for *T* test of normalized log-transformed values. **b** NT-proBNP is freely permeable across the peritoneal membrane, while cNEP is not, as demonstrated by correlation analysis of dialysate with serum or plasma concentrations of NT-proBNP and cNEP; *r* for Pearson’s correlation. *Act.* Activity, *conc.* Concentration, *MW* molecular weight

Mean concentrations of NT-proBNP, GDF-15 and cNEP, as well as cNEP activity by diagnostic group are provided in Table 1 and shown in Fig. 1a. Compared to controls, HF patients demonstrated increased circulating levels of NT-proBNP (*p* < 0.001) and GDF-15 (*p* = 0.014), while cNEP concentration was similar (*p* = 0.818). cNEP activity was significantly lower in HF vs. controls (*p* < 0.001). Of note, controls had noticeably elevated NT-proBNP and GDF-15 levels above the reference range for the general population. In a preliminary study, NT-proBNP, cNEP concentration as well as cNEP activity showed moderate to good correlation before and after HD (Supplementary Fig. 1).

Considering the broad spectrum of molecular weight between NT-proBNP (8.5 kDa), GDF-15 (35 kDa), and soluble cNEP (110 kDa) in a subgroup of patients. We evaluated permeability of the peritoneal membrane for these biomarkers. The peritoneal membrane was easily permeable for the low-molecular-weight NT-proBNP and correlation with serum levels was highly significant (*r* = 0.998, *p* < 0.001), while dialysate detectability and correlation with plasma concentrations were poor for both GDF-15 (*r* = 0.548, *p* = 0.008) and for the relatively high-molecular-weight cNEP (*r* = − 0.194, *p* = 0.134) (Fig. 1b).

### Clinical correlates of biomarkers

Univariate and multivariate clinical correlates of each biomarker in the entire sample are shown in Supplementary Table 2. Independent correlates of higher NT-proBNP were older age, higher dialysis vintage, higher lung comet score, comorbidity, and NYHA class as well as history of valve disease. While GDF-15 and cNEP activity remained correlated to NYHA class in multivariate analysis, there were no correlations for cNEP concentration.

### BIA and echocardiography correlates of biomarkers

In univariate analyses of biomarker correlation with BIA and echocardiographic variables (Table 2), NT-proBNP and cNEP concentration were correlated with ECW:TBW ratio, while GDF-15 and cNEP activity were not. Both NT-proBNP and cNEP activity were correlated with LVEF, while there was at best a moderate trend correlation for GDF-15.

**Table 2:** Bioelectric impedance analysis (BIA) and echocardiography correlates of biomarkers

Characteristic	NT-proBNP		GDF-15		cNEP conc		cNEP act	
	*r*^a^	*P*	*r*^a^	*P*	*r*^a^	*P*	*r*^a^	*P*
Bioelectric impedance analysis								
TBW	− 0.085	0.296	0.004	0.964	− 0.093	0.255	0.003	0.966
ICW	− 0.180	**0.027**	− 0.025	0.759	− 0.132	0.105	0.034	0.674
ECW	0.043	0.597	0.040	0.621	− 0.034	0.675	− 0.036	0.656
ECW:TBW ratio	0.400	** < 0.001**	0.101	0.216	0.168	**0.039**	− 0.130	0.110
OH	0.363	** < 0.001**	0.075	0.360	0.087	0.288	− 0.082	0.318
LTM	− 0.148	0.068	− 0.025	0.756	− 0.139	0.088	0.038	0.645
ATM	− 0.106	0.192	0.000	0.996	0.030	0.711	− 0.012	0.879
Echocardiography								
LVEF	− 0.436	** < 0.001**	− 0.139	0.090	0.014	0.869	0.255	**0.002**
*E*/*e*ʹ	0.486	** < 0.001**	0.132	0.163	− 0.004	0.968	− 0.295	**0.001**
*E*/*A*	0.145	0.132	− 0.091	0.343	0.093	0.333	− 0.024	0.800
LAVI	0.376	** < 0.001**	0.071	0.429	− 0.042	0.639	− 0.140	0.115
IVSED	− 0.029	0.723	0.068	0.412	− 0.009	0.912	0.075	0.367
LVEDD	0.257	**0.001**	− 0.026	0.752	0.068	0.403	− 0.178	**0.029**
LVPWD	0.072	0.386	0.034	0.680	− 0.058	0.483	− 0.056	0.496
LVMI	0.279	**0.001**	0.020	0.813	0.058	0.486	− 0.128	0.124
LVH	0.137	0.092	0.031	0.707	− 0.016	0.847	− 0.052	0.526

^a^Pearson’s or Spearman’s correlation

*act.* activity, *ATM* adipose tissue mass, *cNEP* circulating neprilysin, *conc.* Concentration, *E*/*A* velocity of early to late transmitral inflow, *ECW* extracellular water, *E*/*e*ʹ, early transmitral flow to early medial-mitral annular diastolic velocity, *GDF-15* growth differentiation factor-15, *ICW* intracellular water, *IVSED* interventricular septum thickness at end-diastole, *LAVI* left atrial volume index, *LTM* lean tissue mass, *LVEDD* left ventricular end-diastolic diameter, *LVEF* left ventricular ejection fraction, *LVH* left ventricular hypertrophy, *LVMI* left ventricular mass index, *LVPWD* left ventricular posterior wall thickness at end-diastole, *NT-proBNP* N-terminal pro-B type natriuretic peptide, *OH* overhydration, *TBW* total body water

**Fig. 2:**
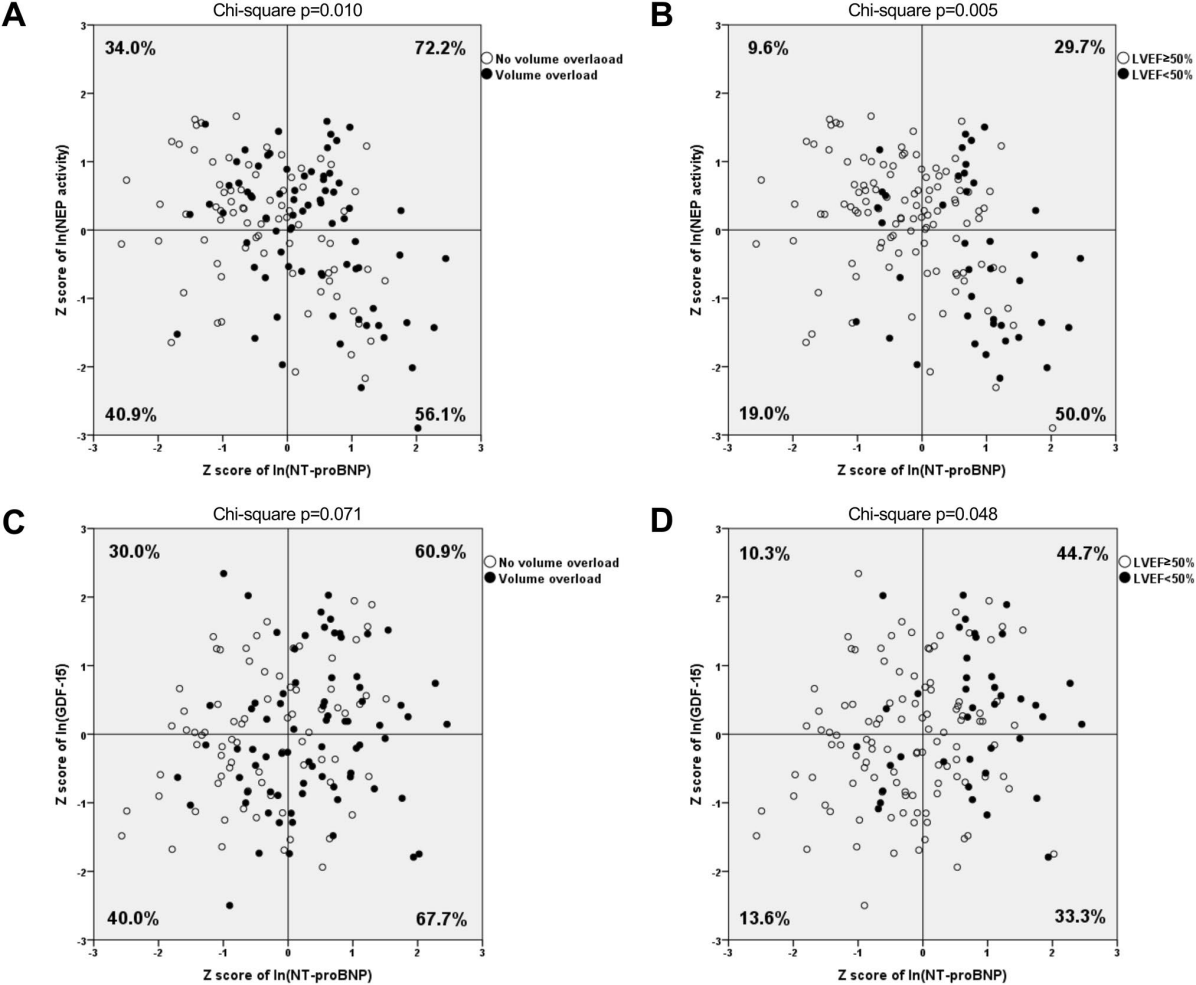
Distribution of volume overload (**a + c**) and systolic dysfunction (LVEF < 50%) (**b** + **d**) according to the circulating concentrations of NT-proBNP and either cNEP activity (**a** + **b**) or GDF-15 (**c** + **d**). Values of NT-proBNP, cNEP activity, and GDF were naturally log-transformed and sex-standardized. Groups were divided into quadrants according to the medians of resulting *Z* scores (represented by lines). Percentages refer to the prevalence of volume overload (**a + c**) and LVEF < 50% (**b + d**) per quadrant

In a further analysis of naturally log-transformed and sex-standardized markers, the prevalence of volume overload was consistently higher with elevated NT-proBNP (56.1–72.2%) compared with patients with low NT-proBNP (34.0–40.9%). Volume overload was most prevalent in patients with increased cNEP activity and NT-proBNP (Fig. 2a, right upper quadrant, Chi-square *p* = 0.010). Similarly, volume overload was more prevalent with elevated NT-proBNP (60.9–67.7%) compared with low NT-proBNP (30.0–40.0%), regardless of GDF-15 (Fig. 2c, right upper and right lower quadrants, Chi-square *p* = 0.071). Furthermore, elevated NT-proBNP and decreased cNEP activity (Fig. 2b, right lower quadrant) demonstrated a 5.2-fold prevalence of LV dysfunction (50.0 vs. 9.6%, *p* = 0.005) compared with low NT-proBNP and high cNEP activity (left upper quadrant). Similarly, both elevated NT-proBNP and GDF-15 (Fig. 2d, right upper quadrant) indicated a 3.3-fold prevalence of LV dysfunction (44.7 vs. 13.6%, *p* = 0.048) compared with low NT-proBNP and GDF-15 (left lower quadrant).

### Diagnostic value of biomarkers and multi-marker models

**Fig. 3:**
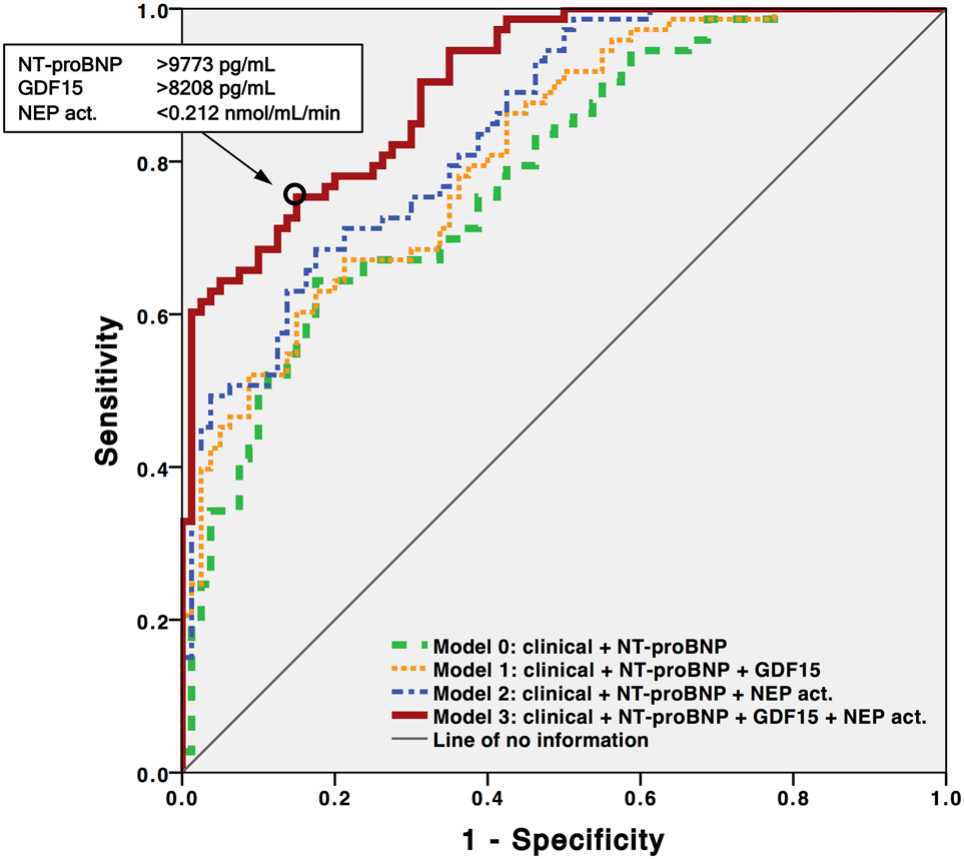
Comparison of areas under the receiver operating characteristics (ROC) curves for prediction of congestive heart failure. Models 0–3 denote the clinical base model supplemented with respective biomarker information, *p* < 0.001 for ROC curves of all models against line of no information. Model 3 demonstrated the largest AUC with 0.902 (95% CI 0.857–0.947) and provided incremental predictive utility over model 0 (*p* < 0.001), model 1 (*p* < 0.001), and model 2 (*p* < 0.001), respectively. Biomarker cutoff values in the insert represent the best relation between sensitivity and specificity (circle). The clinical base model included the following covariates: age, dyspnea score, systolic blood pressure, Charlson comorbidity index, history of congestive heart failure, history of severe valve disease, and extracellular to total body water ratio

In multiple logistic regression analyses of a clinical base model supplemented with NT-proBNP and other multi-marker models, we determined that biomarkers NT-proBNP, GDF-15, and cNEP activity provided strong diagnostic value for HF, while cNEP concentration did not (Table 3). Severe valve disease and previous history of HF provided strongest discrimination between HF and controls. Among models 0–3, a combination of clinical covariates supplemented with information on sex-normalized log-transformed NT-proBNP, GDF-15 and cNEP activity (model 3) provided the best diagnostic value for HF, with an odds ratio of 3.822. In analyses comparing the areas under the curve (AUC) of receiver operating characteristics (ROC) curves (Fig. 3), we show that the addition of biomarkers GDF-15 and cNEP activity, either alone (models 1 and 2) or combined (model 3), increased the combined explanatory power of the base model consisting of clinical covariates and NT-proBNP (model 0): Compared to the base model (AUC_model 0_ = 0.785), we found a significant incremental increase for the diagnostic value of HF with addition of GDF-15 (AUC_model 1_ = 0.814, *p* = 0.015), cNEP activity (AUC_model 2_ = 0.843, *p* < 0.001), and both GDF-15 and cNEP activity (AUC_model 3_ = 0.902, *p* < 0.001), respectively. Model 3 was superior to both models 1 and 2 (p < 0.001 vs. AUC_model 1_ and vs. AUC_model 2_). Subgroup ROC curve analyses demonstrated similar but slightly better discrimination in PD (AUC_model 3_ = 0.923) compared to HD patients (AUC_model 3_ = 0.892). Also, model 3 still delivered best results compared to models 0–2 when distinctly analyzing systolic (AUC_model 3_ = 0.929) or diastolic HF (AUC_model 3_ = 0.869) vs. controls (Supplementary Fig. 2).

**Table 3:** Multiple logistic regression analysis of factors used for differentiating between patients with and those without heart failure

Predictor	OR (95% CI)	*P*
Clinical base model		
Age^a^	1.026 (1.007–1.046)	0.007
Dyspnea score	1.385 (1.095–1.753)	0.007
Systolic BP^a^	1.015 (1.001–1.028)	0.034
Charlson comorbidity index	1.180 (1.050–1.324)	0.005
Previous history of HF	3.470 (1.565–7.696)	0.002
Severe valve disease	4.388 (1.171–16.444)	0.028
ECW:TBW ratio	2.634 (1.367–5.075)	0.004
Model 0^b^: clinical + NT-proBNP	2.505 (1.630–3.847)	< 0.001
Model 1^b^: clinical + NT-proBNP + GDF-15	2.730 (1.801–4.139)	< 0.001
Model 2^b^: clinical + NT-proBNP + cNEP activity	3.010 (1.965–4.613)	< 0.001
Model 3^b^: clinical + NT-proBNP + GDF-15 + cNEP activity	3.822 (2.388–6.117)	< 0.001

^a^ The odds ratio for age and systolic BP represents the exponent for each year of age and each mmHg in the logistic equation, respectively

^b^Models 0–3 denote the clinical base model supplemented with respective information on sex-normalized log-transformed biomarkers

*BP* blood pressure, *cNEP* circulating neprilysin, *CI* confidence interval, *ECW* extracellular water, *GDF-15* growth differentiation factor-15, *HF* congestive heart failure, *NT-proBNP* N-terminal pro-B type natriuretic peptide, *OR* Odds ratio, *TBW* total body water

## Discussion

In this study, we examined the value of NT-proBNP, GDF-15, and cNEP concentration as well as cNEP activity for the diagnosis of HF in ESRD patients on dialysis. A multi-marker model combining clinical covariates, NT-proBNP, GDF-15, and cNEP activity provided excellent discrimination between HF and controls. While NT-proBNP is a well-established biomarker for HF in the general population [5], its applicability is limited in dialysis patients because of decreased renal excretion and volume overload [7, 27]. To yield information from NT-proBNP for LV function in dialysis patients, correction for volume status and using a very high cutoff value have been proposed [7]. Adding biomarkers of distinct mechanisms in HF pathophysiology is another avenue to increase sensitivity and especially specificity for HF diagnosis in ESRD patients.

We show that in addition to NT-proBNP, both GDF-15 and cNEP activity differ significantly between HF and controls. Vodovar et al. showed that the biologically active B-type natriuretic peptide directly inhibits cNEP activity but not cNEP concentration in HF patients [28]. It is known that cNEP activity predicts future hospitalization for HF in CKD patients and that cNEP activity but not cNEP concentration is associated with HF in non-dialysis-dependent CKD patients [20]. We confirm these findings for the first time in a cohort of chronic dialysis patients. Of note, circulating concentrations of NT-proBNP and GDF-15 in controls were raised remarkably above the upper limit of normal for the general population. Elevation of circulating markers due to decreased renal elimination has been demonstrated previously [7, 9, 29]. Therefore, we were interested in dialyzability of all three markers. We show that NT-proBNP permeates freely across the peritoneal membrane in patients on PD due to its low molecular size, while GDF-15 and cNEP did not. However, lack of validation of the latter two markers for peritoneal dialysate cannot be excluded as a reason for poor association of plasma and dialysate values. We found that dialysate NT-proBNP levels were around 1/5 of serum NT-proBNP, confirming what has been found in plasma [30]. Others have shown its presence in dialysate of HD patients and its independence of acute intradialytic events such as hypotension [31]. Therefore, excessively high NT-proBNP levels in controls are most likely elicited by changes in volume status in these patients. Along those lines, we demonstrate that in ESRD patients, NT-proBNP is well correlated with overhydration and different clinical and BIA measures of volume status.

Adding GDF-15 and cNEP activity to a base model (consisting of clinical covariates and NT-proBNP) resulted in a significant increase of the AUC for the diagnostic value of HF from 0.785 to 0.902. When analyzed singularly, however, neither cNEP activity nor GDF-15 yielded satisfactory discrimination between HF and controls. This indicates that cNEP activity and GDF-15 mostly provided additional orthogonal information for HF diagnosis in settings where NT-proBNP discriminated HF vs. controls insufficiently. Given that HF pathophysiology is very complex in ESRD, it seems reasonable that NT-proBNP alone is not adequate to cope with this complexity [32]. One reason for this incremental information of GDF-15 and cNEP activity is that NT-proBNP was strongly correlated to volume overload, while GDF-15 and cNEP activity were not, as indicated by BIA and lung ultrasound assessment. Similarly, there is ample evidence for dependence of NT-proBNP concentration on volume status [33, 34]. In contrast to that, all three biomarkers demonstrated good correlation with LV function. These results indicate that cNEP activity and GDF-15 represent distinct mechanisms of HF pathophysiology and provide additional diagnostic value for HF independent of volume status. As it is known that the prevalence and the dynamics of hypervolemia differ in HD vs. PD patients, in separate sub-analyses we confirmed that our diagnostic model is valid in these two individual patient subgroups with only little differences. Although the algorithm seemed to perform slightly better in PD (AUC_model 3_ = 0.923) compared to HD patients (AUC_model 3_ = 0.892), due to the low sample size we refrained from drawing statistically valid conclusions. Furthermore, we show that model 3 still provided the best discrimination when separately analyzing systolic and diastolic HF, respectively. Slightly lower AUC in diastolic (0.869) vs. systolic HF (0.929) might be due to the fact that the preserved EF in patients suffering from diastolic HF may cause lower levels of the biomarkers studied, possibly leading to slightly less efficient discrimination between controls and HF patients by the multi-marker model.

Our study has several limitations. This was a single-center study with limited sample size. For statistical reasons we did not differentiate between HFrEF, HF with mid-range EF or HFpEF in our analysis [35]. However, we were able to show that analyses for ROC curves yielded nearly similar results when looking distinctly at either systolic or diastolic HF. Echocardiography records were reviewed by a single experienced cardiologist blinded from the clinical data, different cardiologists performed the echocardiography causing potential inter-observer variability. All other clinical examinations were performed by the same observer.

The multi-marker model for diagnosing HF in dialysis patients presented here may be used in a dialysis outpatient context to determine the presence of HF when echocardiography is not promptly available. To the best of our knowledge, we are the first to detect an additional diagnostic benefit of cNEP activity and GDF-15 over clinical covariates and NT-proBNP for HF in the dialysis population. More studies with larger sample size are warranted to confirm our findings and to improve the understanding of HF pathophysiology in dialysis patients. Better mechanistic knowledge of how cNEP activity and GDF-15 provide volume status-independent diagnostic incremental information for HF may facilitate their clinical use in the dialysis population. Finally, biomarker-driven approaches might promote early HF detection in dialysis patients, thereby reducing cardiovascular mortality in this population [36].

## Electronic supplementary material

Below is the link to the electronic supplementary material.

Supplementary file1 (DOCX 22 kb)

Supplementary file2 (PDF 264 kb)

## Abbreviations

ACE-I: Angiotensin-converting enzyme inhibitor
APD: Automated peritoneal dialysis
ARB: Angiotensin receptor blocker
ATM: Adipose tissue mass
AUC: Area under the curve
BIA: Bioelectrical impedance analysis
BMI: Body mass index
BP: Blood pressure
CAPD: Continuous ambulatory peritoneal dialysis
BSA: Body surface area
CABG: Coronary artery bypass graft
CI: Confidence interval
CKD: Chronic kidney disease
cNEP: Circulating neprilysin
COPD: Chronic obstructive pulmonary disease
CRP: C-reactive protein
E/A: Velocity of early to late transmitral inflow
ECW: Extracellular water
E/e’: Early transmitral flow to early medial-mitral annular diastolic velocity
ESRD: End-stage renal disease
GDF-15: Growth differentiation factor-15
GFR: Glomerular filtration rate
HD: Hemodialysis
HDL: High-density lipoprotein
HF: Congestive heart failure
HFpEF: Heart failure with preserved ejection fraction
HFrEF: Heart failure with reduced ejection fraction
ICW: Intracellular water
IQR: Interquartile range
IU: International unit
IVSED: Interventricular septum thickness at end-diastole
LAVI: Left atrial volume index
LDL: Low-density lipoprotein
LTM: Lean tissue mass
LVEF: Left ventricular ejection fraction
LVH: Left ventricular hypertrophy
LVEDD: Left ventricular end-diastolic diameter
LVMI: Left ventricular mass index
LVPWD: Left ventricular posterior wall thickness at end-diastole
MCH: Mean corpuscular hemoglobin
MCHC: Mean corpuscular hemoglobin concentration
MCV: Mean corpuscular volume
MW: Molecular weight
NT-proBNP: N-terminal pro-B-type natriuretic peptide
NYHA: New York Heart Association
OH: Overhydration
OR: Odds ratio
ROC: Receiver operating characteristics
PD: Peritoneal dialysis
PTCA: Percutaneous transluminal coronary angioplasty
PTH: Parathyroid hormone
SD: Standard deviation
TBW: Total body water

## References

[1] Go AS, Chertow GM, Fan D, McCulloch CE, Hsu CY (2004). Chronic kidney disease and the risks of death, cardiovascular events, and hospitalization. N Engl J Med.

[2] Stack AG, Bloembergen WE (2001). A cross-sectional study of the prevalence and clinical correlates of congestive heart failure among incident US dialysis patients. Am J Kidney Dis.

[3] Harnett JD, Foley RN, Kent GM, Barre PE, Murray D, Parfrey PS (1995). Congestive heart failure in dialysis patients: prevalence, incidence, prognosis and risk factors. Kidney Int.

[4] Payne J, Sharma S, De Leon D, Lu JL, Alemu F, Balogun RA, Malakauskas SM, Kalantar-Zadeh K, Kovesdy CP (2012). Association of echocardiographic abnormalities with mortality in men with non-dialysis-dependent chronic kidney disease. Nephrol Dial Transplant.

[5] Maisel AS, Krishnaswamy P, Nowak RM, McCord J, Hollander JE, Duc P, Omland T, Storrow AB, Abraham WT, Wu AH, Clopton P, Steg PG, Westheim A, Knudsen CW, Perez A, Kazanegra R, Herrmann HC, McCullough PA, Breathing Not Properly Multinational Study I (2002). Rapid measurement of B-type natriuretic peptide in the emergency diagnosis of heart failure. N Engl J Med.

[6] Maisel A, Mueller C, Adams K, Anker SD, Aspromonte N, Cleland JG, Cohen-Solal A, Dahlstrom U, DeMaria A, Di Somma S, Filippatos GS, Fonarow GC, Jourdain P, Komajda M, Liu PP, McDonagh T, McDonald K, Mebazaa A, Nieminen MS, Peacock WF, Tubaro M, Valle R, Vanderhyden M, Yancy CW, Zannad F, Braunwald E (2008). State of the art: using natriuretic peptide levels in clinical practice. Eur J Heart Fail.

[7] David S, Kumpers P, Seidler V, Biertz F, Haller H, Fliser D (2008). Diagnostic value of N-terminal pro-B-type natriuretic peptide (NT-proBNP) for left ventricular dysfunction in patients with chronic kidney disease stage 5 on haemodialysis. Nephrol Dial Transplant.

[8] Alehagen U, Lindstedt G, Eriksson H, Dahlstrom U (2003). Utility of the amino-terminal fragment of pro-brain natriuretic peptide in plasma for the evaluation of cardiac dysfunction in elderly patients in primary health care. Clin Chem.

[9] Wollert KC, Kempf T, Wallentin L (2017). Growth differentiation factor 15 as a biomarker in cardiovascular disease. Clin Chem.

[10] Katus HA, Giannitsis E (2018). Biomarker in cardiology: DGK welcomes ESC Munich 2018. Clin Res Cardiol.

[11] Stahrenberg R, Edelmann F, Mende M, Kockskamper A, Dungen HD, Luers C, Binder L, Herrmann-Lingen C, Gelbrich G, Hasenfuss G, Pieske B, Wachter R (2010). The novel biomarker growth differentiation factor 15 in heart failure with normal ejection fraction. Eur J Heart Fail.

[12] Santema BT, Chan MMY, Tromp J, Dokter M, van der Wal HH, Emmens JE, Takens J, Samani NJ, Ng LL, Lang CC, van der Meer P, Ter Maaten JM, Damman K, Dickstein K, Cleland JG, Zannad F, Anker SD, Metra M, van der Harst P, de Boer RA, van Veldhuisen DJ, Rienstra M, Lam CSP, Voors AA (2019). The influence of atrial fibrillation on the levels of NT-proBNP versus GDF-15 in patients with heart failure. Clin Res Cardiol.

[13] Sinning C, Ojeda F, Wild PS, Schnabel RB, Schwarzl M, Ohdah S, Lackner KJ, Pfeiffer N, Michal M, Blettner M, Munzel T, Kempf T, Wollert KC, Kuulasmaa K, Blankenberg S, Salomaa V, Westermann D, Zeller T (2017). Midregional proadrenomedullin and growth differentiation factor-15 are not influenced by obesity in heart failure patients. Clin Res Cardiol.

[14] Tuegel C, Katz R, Alam M, Bhat Z, Bellovich K, de Boer I, Brosius F, Gadegbeku C, Gipson D, Hawkins J, Himmelfarb J, Ju W, Kestenbaum B, Kretzler M, Robinson-Cohen C, Steigerwalt S, Bansal N (2018). GDF-15, galectin 3, soluble ST2, and risk of mortality and cardiovascular events in CKD. Am J Kidney Dis.

[15] Bayes-Genis A, Barallat J, Richards AM (2016). A test in context: neprilysin: function, inhibition, and biomarker. J Am Coll Cardiol.

[16] McMurray JJ, Packer M, Desai AS, Gong J, Lefkowitz MP, Rizkala AR, Rouleau JL, Shi VC, Solomon SD, Swedberg K, Zile MR, Investigators P-H, Committees (2014). Angiotensin-neprilysin inhibition versus enalapril in heart failure. N Engl J Med.

[17] Bayes-Genis A, Barallat J, Pascual-Figal D, Nunez J, Minana G, Sanchez-Mas J, Galan A, Sanchis J, Zamora E, Perez-Martinez MT, Lupon J (2015). Prognostic value and kinetics of soluble neprilysin in acute heart failure: a pilot study. JACC Heart Fail.

[18] Goliasch G, Pavo N, Zotter-Tufaro C, Kammerlander A, Duca F, Mascherbauer J, Bonderman D (2016). Soluble neprilysin does not correlate with outcome in heart failure with preserved ejection fraction. Eur J Heart Fail.

[19] Bayes-Genis A, Barallat J, Galan A, de Antonio M, Domingo M, Zamora E, Gastelurrutia P, Vila J, Penafiel J, Galvez-Monton C, Lupon J (2015). Multimarker strategy for heart failure prognostication Value of neurohormonal biomarkers: neprilysin vs NT-proBNP. Rev Esp Cardiol (Engl Ed).

[20] Emrich IE, Vodovar N, Feuer L, Untersteller K, Nougue H, Seiler-Mussler S, Fliser D, Launay JM, Heine GH (2019). Do plasma neprilysin activity and plasma neprilysin concentration predict cardiac events in chronic kidney disease patients?. Nephrol Dial Transplant.

[21] Kempf T, Horn-Wichmann R, Brabant G, Peter T, Allhoff T, Klein G, Drexler H, Johnston N, Wallentin L, Wollert KC (2007). Circulating concentrations of growth-differentiation factor 15 in apparently healthy elderly individuals and patients with chronic heart failure as assessed by a new immunoradiometric sandwich assay. Clin Chem.

[22] Nougue H, Pezel T, Picard F, Sadoune M, Arrigo M, Beauvais F, Launay JM, Cohen-Solal A, Vodovar N, Logeart D (2018). Effects of sacubitril/valsartan on neprilysin targets and the metabolism of natriuretic peptides in chronic heart failure: a mechanistic clinical study. Eur J Heart Fail.

[23] Kataoka H, Matsuno O (2008). Age-related pulmonary crackles (rales) in asymptomatic cardiovascular patients. Ann Fam Med.

[24] Torino C, Gargani L, Sicari R, Letachowicz K, Ekart R, Fliser D, Covic A, Siamopoulos K, Stavroulopoulos A, Massy ZA, Fiaccadori E, Caiazza A, Bachelet T, Slotki I, Martinez-Castelao A, Coudert-Krier MJ, Rossignol P, Gueler F, Hannedouche T, Panichi V, Wiecek A, Pontoriero G, Sarafidis P, Klinger M, Hojs R, Seiler-Mussler S, Lizzi F, Siriopol D, Balafa O, Shavit L, Tripepi R, Mallamaci F, Tripepi G, Picano E, London GM, Zoccali C (2016). The agreement between auscultation and lung ultrasound in hemodialysis patients: the LUST study. Clin J Am Soc Nephrol.

[25] Nagueh SF, Smiseth OA, Appleton CP, Byrd BF, Dokainish H, Edvardsen T, Flachskampf FA, Gillebert TC, Klein AL, Lancellotti P, Marino P, Oh JK, Popescu BA, Waggoner AD (2016). Recommendations for the evaluation of left ventricular diastolic function by echocardiography: an update from the American Society of echocardiography and the European Association of cardiovascular imaging. J Am Soc Echocardiogr.

[26] Wang TJ, Gona P, Larson MG, Tofler GH, Levy D, Newton-Cheh C, Jacques PF, Rifai N, Selhub J, Robins SJ, Benjamin EJ, D'Agostino RB, Vasan RS (2006). Multiple biomarkers for the prediction of first major cardiovascular events and death. N Engl J Med.

[27] Locatelli F, Pozzoni P, Tentori F, del Vecchio L (2003). Epidemiology of cardiovascular risk in patients with chronic kidney disease. Nephrol Dial Transplant.

[28] Vodovar N, Seronde MF, Laribi S, Gayat E, Lassus J, Januzzi JL, Boukef R, Nouira S, Manivet P, Samuel JL, Logeart D, Cohen-Solal A, Richards AM, Launay JM, Mebazaa A (2015). Elevated plasma B-type natriuretic peptide concentrations directly inhibit circulating neprilysin activity in heart failure. JACC Heart Fail.

[29] Luchner A, Hengstenberg C, Lowel H, Riegger GA, Schunkert H, Holmer S (2005). Effect of compensated renal dysfunction on approved heart failure markers: direct comparison of brain natriuretic peptide (BNP) and N-terminal pro-BNP. Hypertension.

[30] Koz S, Sahin I, Temel I, Koz ST, Terzi Z (2016). Elimination of NTproBNP in peritoneal dialysis patients Does peritoneal membrane type make a difference in plasma level and elimination of NTproBNP?. Clin Nephrol.

[31] Wahl HG, Graf S, Renz H, Fassbinder W (2004). Elimination of the cardiac natriuretic peptides B-type natriuretic peptide (BNP) and N-terminal proBNP by hemodialysis. Clin Chem.

[32] Tuegel C, Bansal N (2017). Heart failure in patients with kidney disease. Heart.

[33] Booth J, Pinney J, Davenport A (2010). N-terminal proBNP–marker of cardiac dysfunction, fluid overload, or malnutrition in hemodialysis patients?. Clin J Am Soc Nephrol.

[34] Papakrivopoulou E, Lillywhite S, Davenport A (2012). Is N-terminal probrain-type natriuretic peptide a clinically useful biomarker of volume overload in peritoneal dialysis patients?. Nephrol Dial Transplant.

[35] Ponikowski P, Voors AA, Anker SD, Bueno H, Cleland JG, Coats AJ, Falk V, Gonzalez-Juanatey JR, Harjola VP, Jankowska EA, Jessup M, Linde C, Nihoyannopoulos P, Parissis JT, Pieske B, Riley JP, Rosano GM, Ruilope LM, Ruschitzka F, Rutten FH, van der Meer P (2016). 2016 ESC Guidelines for the diagnosis and treatment of acute and chronic heart failure: the task force for the diagnosis and treatment of acute and chronic heart failure of the European Society of Cardiology (ESC) Developed with the special contribution of the Heart Failure Association (HFA) of the ESC. Eur J Heart Fail.

[36] Mueller C, McDonald K, de Boer RA, Maisel A, Cleland JGF, Kozhuharov N, Coats AJS, Metra M, Mebazaa A, Ruschitzka F, Lainscak M, Filippatos G, Seferovic PM, Meijers WC, Bayes-Genis A, Mueller T, Richards M, Januzzi JL, Jr., Heart Failure Association of the European Society of C (2019). Heart failure association of the European Society of cardiology practical guidance on the use of natriuretic peptide concentrations. Eur J Heart Fail.

