# Supplementary material for: Soluble neprilysin, NT-proBNP, and growth differentiation factor-15 as biomarkers for heart failure in dialysis patients (SONGBIRD)
Source: Clin Res Cardiol. 2020 Jan 30;109(8):1035–47. doi: 10.1007/s00392-020-01597-x (PMC7376515; doi:10.1007/s00392-020-01597-x)
Supplement: Supplementary file 1 — Supplementary file1 (DOCX 22 kb) [file 392_2020_1597_MOESM1_ESM.docx]

**SUPPLEMENTARY MATERIAL**

**figure legends**

**Supplementary Fig. 1** Correlations of pre and post HD NT-proBNP (**A**), cNEP concentration (**B**), and cNEP activity (**C)**, respectively.

**Supplementary Fig. 2** Comparison of areas under the ROC curves for HF diagnosis in subgroups HD (**A**) and PD (**B**). Note that model 3 also shows best discrimination for both systolic (**C**) and diastolic (**D**) HF, when analyzed separately. Models 0 to 3 denote the clinical base model supplemented with respective biomarker information, p<0.001 for ROC curves of all models against line of no information. The clinical base model included the following covariates: age, dyspnea score, systolic blood pressure, Charlson comorbidity index, history of congestive heart failure, history of severe valve disease, and extracellular to total body water ratio.

**Supplementary Table 1** Comorbidity, medication and bioelectrical impedance analysis, echocardiography and laboratory patient characteristics

| Characteristic | |  | Controls  (N=80) | HF  (N=73) | P |
| --- | --- | --- | --- | --- | --- |
| *Comorbidity* | |  |  |  |  |
| Charlson comorbidity score (mean±SD) | |  | 5.6±2.9 | 7.0±2.8 | **0.002** |
| Myocardial infarction, n (%) | |  | 13 (16.3) | 15 (20.5) | 0.535 |
| Peripheral vascular disease, n (%) | |  | 11 (13.8) | 17 (23.3) | 0.146 |
| Cerebrovascular accident/transient ischemic attack, n (%) | |  | 15 (18.8) | 13 (17.8) | 1.000 |
| COPD, n (%) | |  | 10 (12.5) | 13 (17.8) | 0.376 |
| Connective tissue disease, n (%) | |  | 8 (10.0) | 7 (9.6) | 1.000 |
| Peptic ulcer disease, n (%) | |  | 9 (11.3) | 12 (16.4) | 0.481 |
| Liver disease, n (%) | |  |  |  | 0.512 |
| Mild | |  | 16 (20.0) | 20 (27.4) |  |
| Moderate to severe | |  | 2 (2.5) | 1 (1.4) |  |
| Diabetes mellitus, n (%) | |  |  |  | 0.417 |
| Without end organ damage | |  | 7 (8.8) | 8 (11.0) |  |
| With end organ damage | |  | 20 (25.0) | 12 (16.4) |  |
| Malignancy, n (%) | |  |  |  | **0.028** |
| M0 | |  | 9 (11.3) | 20 (27.4) |  |
| M1 | |  | 1 (1.3) | 2 (2.7) |  |
| Coronary artery disease, n (%) | |  | 25 (31.3) | 30 (41.1) | 0.239 |
| PTCA, stent or CABG, n (%) | |  | 16 (20.0) | 18 (24.7) | 0.561 |
| Severe valve disease, n (%) | |  | 3 (3.9) | 11 (15.3) | **0.019** |
| Atrial fibrillation, n (%) | |  | 12 (15.0) | 20 (27.4) | 0.074 |
| Hypertension, n (%) | |  | 74 (92.5) | 69 (94.5) | 0.748 |
| Dyslipidemia, n (%) | |  | 41 (51.2) | 44 (60.3) | 0.329 |
| Active smoker, n (%) | |  | 14 (17.5) | 12 (16.4) | 1.000 |
| Pack years (mean±SD) | |  | 14.0±17.4 | 10.2±16.0 | 0.115 |
| *Medication* | |  |  |  |  |
| Diuretic, n (%) | |  | 51 (63.7) | 40 (54.8) | 0.323 |
| ACE-I/ARB, n (%) | |  | 50 (62.5) | 51 (69.9) | 0.394 |
| Beta blocker, n (%), n (%) | |  | 57 (71.3) | 58 (79.5) | 0.266 |
| Calcium channel blocker, n (%) | |  | 25 (31.3) | 33 (45.2) | 0.095 |
| Other antihypertensive, n (%) | |  | 20 (25.0) | 22 (30.1) | 0.587 |
| Statin, n (%) | |  | 37 (46.3) | 36 (49.3) | 0.747 |
| Platelet inhibitor, n (%) | |  | 38 (47.5) | 37 (50.7) | 0.747 |
| Oral anticoagulation, n (%) | |  | 11 (13.8) | 17 (23.3) | 0.146 |
| Nitrate, n (%) | |  | 2 (2.5) | 4 (5.5) | 0.426 |
| Erythropoetin IU/week (mean±SD) | |  | 7580±6656 | 9389±7445 | 0.141 |
| *Bioelectrical impedance analysis* | |  |  |  |  |
| TBW, L (mean±SD) | |  | 36.3±8.7 | 34.7±8.2 | 0.254 |
| ICW, L (mean±SD) | |  | 19.3±5.1 | 17.7±4.7 | **0.039** |
| ECW, L (mean±SD) | |  | 17.0±4.1 | 17.0±4.0 | 0.859 |
| ECW:TBW ratio (mean±SD) | |  | 0.47±0.04 | 0.49±0.04 | **0.001** |
| OH, L (mean±SD) | |  | 0.65±1.79 | 1.71±2.32 | **0.015** |
| LTM, kg (mean±SD) | |  | 38.6±12.1 | 35.3±11.6 | 0.063 |
| ATM, kg (mean±SD) | |  | 37.4±19.3 | 34.7±15.1 | 0.576 |
| *Echocardiography* | |  |  |  |  |
| LVEF, % (mean±SD) | |  | 56.1±3.7 | 48.3±10.2 | <**0.001** |
| E/e’ | |  | 9.0±3.3 | 16.4±7.0 | <**0.001** |
| E/A | |  | 1.0±0.5 | 1.1±0.5 | 0.957 |
| LAVI, mL/m^2^ (mean±SD) | |  | 37.8±13.6 | 54.1±22.1 | <**0.001** |
| IVSED, cm (mean±SD) | |  | 1.17±0.24 | 1.17±0.26 | 0.856 |
| LVEDD, cm (mean±SD) | |  | 4.72±0.66 | 5.12±0.77 | **0.002** |
| LVPWD, cm (mean±SD) | |  | 1.11±0.20 | 1.16±0.26 | 0.179 |
| LVMI, g/m² (mean±SD) | |  | 106.0±29.1 | 129.7±39.0 | **<0.001** |
| LVH, n (%) | |  | 33 (41.3) | 51 (69.9) | **<0.001** |
| *Laboratory* | |  |  |  |  |
| Sodium, mmol/L (mean±SD) | |  | 139±3.0 | 138±3.0 | 0.061 |
| Potassium, mmol/L (mean±SD) | |  | 4.7±0.5 | 4.7±0.6 | 0.763 |
| Calcium, mmol/L (mean±SD) | |  | 2.13±0.19 | 2.15±0.16 | 0.581 |
| Phosphate, mmol/L (mean±SD) | |  | 1.02±0.54 | 1.02±0.62 | 0.797 |
| PTH, pg/mL (mean±SD) | |  | 256±246 | 235±221 | 0.701 |
| Albumin, g/L (mean±SD) | |  | 35.8±6.4 | 34.7±6.5 | 0.373 |
| CRP, mg/L (mean±SD) | |  | 31.3±49.5 | 33.9±56.0 | 0.437 |
| Glucose, mmol/L (mean±SD) | |  | 6.8±2.3 | 6.8±2.2 | 0.991 |
| Cholesterol, mmol/L (mean±SD) | |  | 3.94±1.11 | 4.12±1.28 | 0.359 |
| LDL, mmol/L (mean±SD) | |  | 2.17±0.93 | 2.39±1.08 | 0.233 |
| HDL, mmol/L (mean±SD) | |  | 1.12±0.42 | 1.22±0.47 | 0.205 |
| Ferritin, µg/L (mean±SD) | |  | 772±891 | 944±943 | 0.160 |
| Transferrin, µmol/L (mean±SD) | |  | 35.8±10.4 | 34.8±9.67 | 0.570 |
| Leucocytes, k/µL (mean±SD) | |  | 7.2±3.0 | 6.8±2.9 | 0.408 |
| Erythrocytes, M/µL (mean±SD) | |  | 3.6±0.6 | 3.4±0.6 | 0.062 |
| MCV, fL (mean±SD) | |  | 91.0±6.4 | 93.0±6.9 | 0.082 |
| MCH, pg (mean±SD) | |  | 32.3±2.6 | 33.1±1.7 | 0.343 |
| MCHC, g/dL (mean±SD) | |  | 31.6±2.2 | 31.4±2.5 | 0.536 |
| Hematocrit, % (mean±SD) | |  | 32.5±4.9 | 31.6±5.0 | 0.285 |
| Hemoglobin, g/dL (mean±SD) | |  | 10.9±1.7 | 10.5±1.7 | 0.234 |
| Platelets, k/µL (mean±SD) | |  | 232±82 | 217±81 | 0.271 |
| ACE-I, angiotensin-converting enzyme inhibitor; ARB, angiotensin receptor blocker, ATM, adipose tissue mass; CABG, coronary artery bypass graft; COPD, chronic obstructive pulmonary disease; CRP, C-reactive protein; E/A, velocity of early to late transmitral inflow; ECW, extracellular water; E/e’ early transmitral flow to early medial-mitral annular diastolic velocity; HDL, high-density lipoprotein; HF, congestive heart failure; ICW, intracellular water; IU, international unit; IVSED, interventricular septum thickness at end-diastole; LAVI, left atrial volume index; LDL, low-density lipoprotein; LTM, lean tissue mass; LVEF, left ventricular ejection fraction; LVH, left ventricular hypertrophy; LVEDD, left ventricular end-diastolic diameter; LVMI, left ventricular mass index; LVPWD, left ventricular posterior wall thickness at end-diastole; MCH, mean corpuscular hemoglobin; MCHC, mean corpuscular hemoglobin concentration; MCV, mean corpuscular volume; OH, overhydration; PTCA, percutaneous transluminal coronary angioplasty; PTH, parathyroid hormone; TBW, total body water. | | | | |  |

**Supplementary Table 2** Clinical correlates of biomarkers

| Characteristic |  | NT-proBNP | | GDF15 | | cNEP conc. | | cNEP act. | |
| --- | --- | --- | --- | --- | --- | --- | --- | --- | --- |
|  |  | r^a^ | Coefficient^b^ (95% CI) | r^a^ | Coefficient^b^ (95% CI) | r^a^ | Coefficient^b^ (95% CI) | r^a^ | Coefficient^b^ (95% CI) |
| *Clinical* |  |  |  |  |  |  |  |  |  |
| Age |  | 0.265* | -0.002 (-0.515 to 0.011) | 0.217* | 0.014 (-0.003 to 0.030) | 0.045 | 0.001 (-0.016 to 0.018) | -0.157 | 0.000 (-0.017 to 0.017) |
| Gender, male^c^ |  | -0.031 | 0.110 (-0.188 to 0.408) | 0.009 | 0.150 (-0.227 to 0.528) | 0.041 | 0.050 (-0.338 to 0.438) | -0.002 | -0.122 (-0.509 to 0.266) |
| Systolic BP |  | 0.138 | 0.005 (-0.001 to 0.012) | 0.020 | -0.002 (-0.010 to 0.006) | -0.151 | -0.004 (-0.012 to 0.004) | -0.138 | -0.004 (-0.012 to 0.004) |
| PD vs. HD^d^ |  | -0.161* | -0.003 (-0.337 to 0.371) | -0.054 | 0.183 (-0.291 to 0.657) | -0.143 | -0.093 (-0.580 to 0.394) | 0.041 | -0.061 (-0.548 to 0.426) |
| Dialysis vintage |  | 0.252* | 0.001 (-0.001 to 0.004) | 0.214* | 0.003 (0.000 to 0.006) | 0.061 | 0.001 (-0.002 to 0.004) | -0.046 | 0.000 (-0.003 to 0.003) |
| Net ultrafiltration |  | 0.239* | 0.394 (0.114 to 0.674)* | 0.003 | 0.073 (-0.281 to 0.428) | 0.012 | -0.196 (-0.561 to 0.168) | -0.123 | -0.171 (-0.535 to 0.193) |
| Volume status score |  | 0.215* | 0.001 (-0.171 to 0.174) | 0.148 | 0.084 (-0.134 to 0.302) | 0.013 | 0.001 (-0.223 to 0.225) | -0.195* | -0.127 (-0.351 to 0.097) |
| Vena cava inferior diameter |  | 0.227* | -0.019 (-0.051 to 0.013) | 0.046 | -0.022 (-0.062 to 0.019) | 0.042 | 0.005 (-0.036 to 0.047) | -0.091 | 0.002 (-0.040 to 0.044) |
| Lung comet score |  | 0.466* | 0.009 (0.000 to 0.017)* | 0.205* | 0.010 (-0.001 to 0.020) | -0.066 | -0.006 (-0.016 to 0.005) | -0.140 | -0.002 (-0.012 to 0.009) |
| *Comorbidity* |  |  |  |  |  |  |  |  |  |
| Charlson comorbidity score |  | 0.290* | -0.032 (-0.111 to 0.047) | 0.184* | -0.040 (-0.139 to 0.060) | 0.088 | -0.030 (-0.132 to 0.073) | -0.130 | -0.036 (-0.394 to 0.067) |
| NYHA class |  | 0.509* | 0.239 (0.114 to 0.363)* | 0.303* | 0.200 (0.043 to 0.358)* | 0.020 | -0.020 (-0.182 to 0.142) | -0.253* | -0.232 (-0.237 to -0.070)* |
| Coronary artery disease |  | 0.240* | 0.223 (-0.102 to 0.548) | 0.152 | 0.075 (-0.337 to 0.487) | 0.019 | -0.036 (-0.460 to 0.387) | -0.045 | 0.186 (-0.174 to 0.609) |
| Valve disease |  | 0.376* | 0.376 (0.061 to 0.692)* | 0.175* | 0.061 (-0.338 to 0.460) | 0.085 | 0.218 (-0.193 to 0.628) | -0.112 | 0.062 (-0.348 to 0.472) |
| Hypertension |  | 0.198* | 0.394 (-0.242 to 1.031) | 0.053 | 0.337 (-0.470 to 1.143) | -0.082 | 0.046 (-0.783 to 0.874) | -0.151 | -0.450 (-1.278 to 0.378) |
| *Medication* |  |  |  |  |  |  |  |  |  |
| ACE-I/ARB |  | -0.020 | -0.057 (-0.397 to 0.283) | 0.001 | -0.035 (-0.466 to 0.396) | -0.128 | -0.236 (-0.678 to 0.207) | 0.081 | 0.274 (-0.168 to 0.716) |
| Beta blocker |  | 0.178* | 0.203 (-0.123 to 0.529) | -0.012 | -0.130 (-0.543 to 0.283) | -0.060 | -0.042 (-0.466 to 0.383) | -0.109 | -0.181 (-0.605 to 0.243) |
| Oral anticoagulation |  | 0.208* | 0.305 (-0.084 to 0.693) | 0.014 | -0.443 (-0.935 to 0.050) | 0.111 | 0.073 (-0.433 to 0.579) | -0.057 | -0.080 (-0.585 to 0.426) |
| Nitrate |  | 0.170* | 0.376 (-0.314 to 1.065) | 0.035 | -0.548 (-1.421 to 0.326) | 0.034 | 0.079 (-0.818 to 0.976) | -0.033 | 0.159 (-0.737 to 1.056) |
| Erythropoetin dose |  | 0.345* | 0.311 (0.005 to 0.617)* | -0.009 | -0.066 (-0.453 to 0.321) | 0.058 | 0.087 (-0.311 to 0.485) | -0.073 | -0.002 (-0.400 to 0.396) |

^a^ Univariate Pearson’s or Spearman’s correlation; ^b^ multiple linear regression; ^c^ female is the referent group; ^d^ HD is the referent group; * p<0.05; ACE-I, angiotensin-converting enzyme inhibitor; act., activity; ARB, angiotensin receptor blocker; BP, blood pressure; CI, confidence interval; cNEP, circulating neprilysin; conc., concentration; GDF15, growth differentiation factor-15; HD, hemodialysis; NT-proBNP, N-terminal pro-B type natriuretic peptide; NYHA, New York Heart Association; PD, peritoneal dialysis.
