# Supplementary material for: Soluble neprilysin, NT-proBNP, and growth differentiation factor-15 as biomarkers for heart failure in dialysis patients (SONGBIRD)
Source: Clin Res Cardiol. 2020 Jan 30;109(8):1035–47. doi: 10.1007/s00392-020-01597-x (PMC7376515; doi:10.1007/s00392-020-01597-x)

**Supplementary Figure 1.** Correlations of pre and post HD NT-proBNP (**A**), NEP concentration (**B**), and NEP activity (**C)**, respectively.

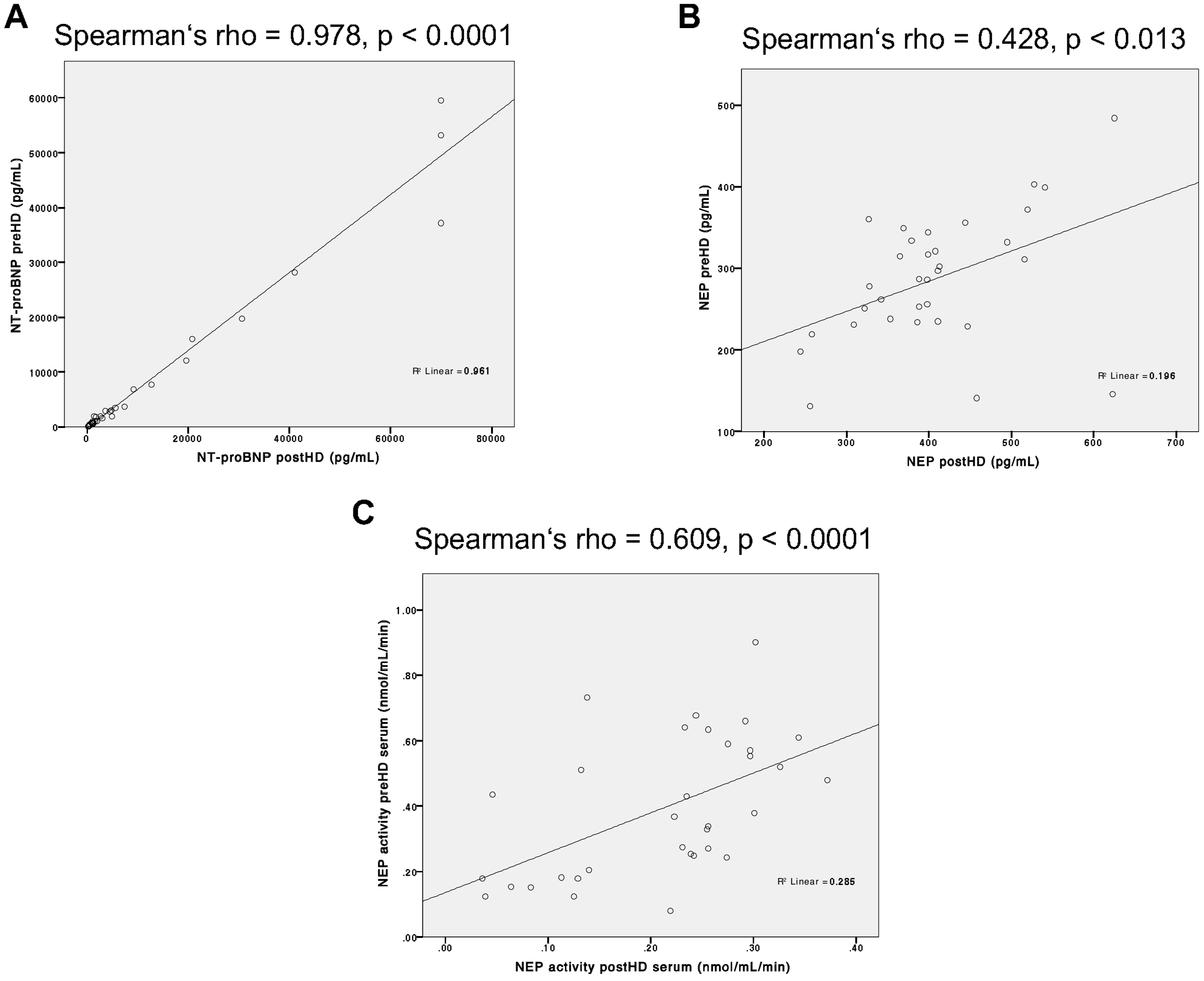

**Supplementary Figure 2.** Comparison of areas under the ROC curves for HF diagnosis in subgroups HD (**A**) and PD (**B**). Note that model 3 also shows best discrimination for both systolic (**C**) and diastolic (**D**) HF, when analyzed separately.

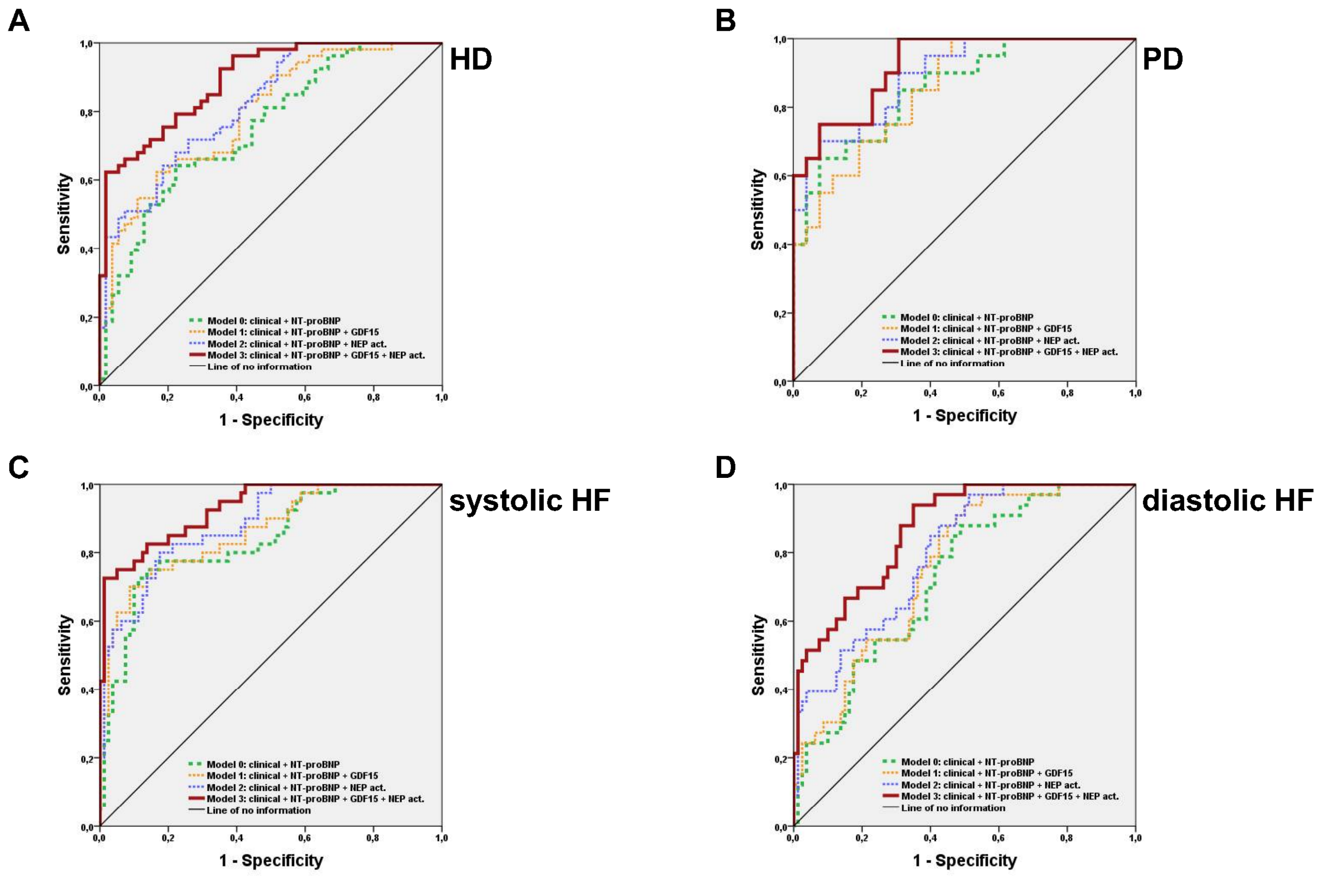

Supplement: Supplementary file 2 — Supplementary file2 (PDF 264 kb) [file 392_2020_1597_MOESM2_ESM.pdf]
